# General Roadmap and Core Steps for the Development of AI Tools in Digital Pathology

**DOI:** 10.3390/diagnostics12051272

**Published:** 2022-05-20

**Authors:** Yasmine Makhlouf, Manuel Salto-Tellez, Jacqueline James, Paul O’Reilly, Perry Maxwell

**Affiliations:** 1 Precision Medicine Centre of Excellence, PathLAKE Programme, The Patrick G Johnston Centre for Cancer Research, Queen’s University Belfast, Belfast BT9 7AE, UK; m.salto-tellez@qub.ac.uk (M.S.-T.); j.james@qub.ac.uk (J.J.); p.maxwell@qub.ac.uk (P.M.); 2 Division of Molecular Pathology, The Institute of Cancer Research, Sutton SM2 5NG, UK; 3 Northern Ireland Biobank, The Patrick G Johnston Centre for Cancer Research, Queen’s University Belfast, Belfast BT9 7AE, UK; 4 Sonrai Analytics Ltd., Lisburn Road, Belfast BT9 7BL, UK; p.oreilly@sonraianalytics.com

**Keywords:** artificial intelligence, digital pathology, human-AI interaction, diagnostic, machine learning, deep learning

## Abstract

Integrating artificial intelligence (AI) tools in the tissue diagnostic workflow will benefit the pathologist and, ultimately, the patient. The generation of such AI tools has two parallel and yet interconnected processes, namely the definition of the pathologist’s task to be delivered *in silico*, and the software development requirements. In this review paper, we demystify this process, from a viewpoint that joins experienced pathologists and data scientists, by proposing a general pathway and describing the core steps to build an AI digital pathology tool. In doing so, we highlight the importance of the collaboration between AI scientists and pathologists, from the initial formulation of the hypothesis to the final, ready-to-use product.

## 1. Introduction

There is a drive in pathology and other disciplines to develop for clinical utility, Artificial intelligence (AI) tools, in order to automate evaluation [1] from large data caches [2]. Such drives are recognized by governments (e.g., The Topol Review by the UK Government, 2019) in response to over 50 years of development in radiology [3,4] and pathology [5,6]. Mimicking routine pathology workflows, technology challenges such as been demonstrated [7], have demonstrated equivalence or improvement in performance by some deep learning networks.

Histological data in digital pathology [8] presents some of the most difficult challenges for achieving either an automated diagnosis or assisting in a diagnosis. Multiple interactive tools have been proposed to aid medical users to automate whole-slide image (WSI) analysis without coding, covering domains of phenotype analysis [9], segmentation [10], and IHC screening [11] This takes several forms, the principal of which is the automated interpretation of pathological images. AI is underpinned by computer algorithms that interrogate the image pixels and quantitatively map them to predefined classes, which represent tissue structures or disease states [2]. Recent research [12,13,14,15] suggests that the design of a diagnostic tool or algorithm often needs to consider how that tool is used, how it fits into a pathologist’s established workflow, and other domain-specific behaviors.

Whether the AI algorithm relies on fully supervised or weakly supervised/unsupervised learning, the main goal of ML algorithms is then to find the best possible mapping between features values and the desired result (a classification, a regression model, a set of clusters, etc.) by searching for patterns in data [16]. In addition to that, there will always be need for input from a pathologist. As an example, the authors in [4] trained a classification model in a weakly supervised manner, using the slide-level diagnosis, which is readily available from anatomic pathology laboratory information systems (LISs) or electronic health records. In addition to the cost of having to use a dataset of 44,732 cases, diagnostic data retrieved from pathology reports is still required. To be more specific, the diagnosis of a case is based on the diagnosis of the slide—the slide level label. This however, can be weak in terms of reliability [5] due to inter-observer discordance, and the use of differences in disease coding e.g., SNOMED code [17], and billing code practice. Standardized electronic reporting formats and comments on suboptimal specimen quality, which requires important input from the pathologist [18]. AI algorithms based on supervised methods strongly rely on 51 pathologist expertise through the provision of a ground truth by which comparison can be made. This therefore, provides robust primary image datasets for AI model development and automation. A weakly-supervised model relies more on the pre-labeled dataset with all of the disadvantages highlighted above. Supervised learning therefore, is more time-consuming and costly to obtain. A trade-off should be considered, between robust, scalable, and reproducible interaction, annotation workflows involving pathologists, and data scientists or larger datasets used in unsupervised, self-supervised, or multiple instance learning approaches to AI. Although supervised learning remains the state of the art in terms of accuracy metrics in image processing, recent results have shown that unsupervised and weakly-supervised learning can achieve creditable results in image processing tasks [19]. This approach can recognize more detailed features that the pathologist may not easily find, allowing sometimes a better way to subtype diseases. In the pathology domain, these approaches have the potential to reduce the need for annotations, but still require input from pathologists to improve reliability and for validation.

While the field of digital pathology has recently witnessed a surge in publications describing the state-of-the-art performance for machine learning models across a wide range of diagnostic applications [20], only a few papers describe the collaborative work and exchange between the AI scientists and pathologists to build these applications. Given the importance of the interactional relationship between pathologists and AI scientists [20], and the need for robust, scalable, and reproducible interaction and annotation workflows, we propose a general workflow. This viewpoint is based on the experiences of experimented pathologists and AI scientists, and describes the main building blocks for developing a tool in pathology with clinical utility, summarized as follows:

- Hypothesis formulation.
- Data preparation in the wet-lab and digital annotations and pre-processing.
- AI models design, training, and clinical validation.
- Deployment.

We also highlight how AI scientists and pathologists collaborate at each of these steps, from hypothesis formulation to the deployment tool.

## 2. Workflow and Methods

### 2.1. Hypothesis Formulation

The clinical utility of any AI tool requires definition, and resultant hypothesis formulation by a pathologist to deliver an AI tool that seeks to deliver or facilitate diagnostic clinical output. This has been defined in the background and rationale section of the SPIRIT-AI extension to the SPIRIT 2013 protocol for clinical trials [21], which specifically seeks to address clinical interventions, which have an AI component. Given that the final goal of many algorithms in development is the implementation in a clinical pathway, algorithm developers should be aware of such concerns. Specific objectives should be set, such as a clear process based on gathering information from the slide, performing a series of tests and estimations, and finally arriving, for example, at a diagnosis. As an example, a pathologist may rely on the density estimation of a specific biomarker to analyze its effect on a patient’s outcome and recommends a treatment pathway from which the patient is likely to benefit. A clear hypothesis formulation helps AI scientists to identify the type of image analysis tools and the most appropriate category of algorithms required to solve the stated problem.

### 2.2. Wet-Lab Work

Once pathologists formulate the hypothesis, the dataset source in the form of slides needs to be established either through existing archives or through the staining during the wet lab process. The method used to stain tissues influences the type of image analysis that can be performed [22]. Hematoxylin and Eosin (H&E) staining is a common histochemical stain used to evaluate a tissue’s morphology [22]. Another methodology is immunohistochemistry (IHC) staining for labeling tissues for specific proteins as biomarkers, where primary antibodies are used to bind to the epitope(s) of interest within the tissue section, and secondary antibodies detect these proteins, their binding made visible enabling signal amplification and visualization. If it is necessary to identify several proteins in or on the same cell (e.g., phenotyping immune cell populations and assessing spatial relationships among various cell types). One approach would be the consecutive serial sections of single-plex IHC staining followed by digital image registration, alignment, and fusion into a single plane. Another approach is multiplex staining to detect several proteins within a single tissue section. While the former approach uses more tissue, the latter can pose significant challenges in the development of appropriate staining protocols [22].

### 2.3. Data Preparation

#### 2.3.1. Quality Check

**Figure 1:**
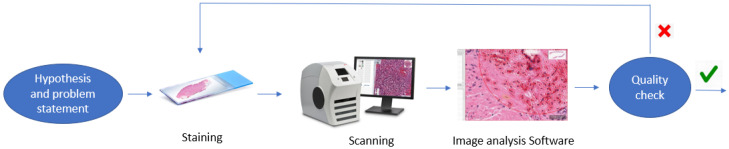
From hypothesis to slides validation for image processing.

The pathologist works on the preparation of input data that needs to be presented to the AI to allow it to serve its purpose. This is done through eligibility criteria stating the inclusion and exclusion criteria at the level of both participants and the input data [21]. The pathologist also checks the quality of the slides after the staining process in the wet lab, followed by an assessment of whether a slide should be included in the dataset used to develop the AI tool. The pathologist may also ask for a repeat of the scanning process or suggest another staining approach that may improve the quality of the slide. Once this is done, the initial dataset, in its digitized format, is passed to the AI scientists for further processing. Figure 1 illustrates this process from hypothesis to slide validation.

#### 2.3.2. Descriptive Region

In addition to the quality check, and depending on the problem at hand, the pathologist can suggest a region of interest within the slide that can be more descriptive for the patient diagnostic and/or any other desired outcome.

#### 2.3.3. Annotations

AI models learn from examples, and AI scientists rely on a pathologist’s knowledge and expertise to provide them with these examples. It should begin with specifying the procedure for acquiring and selecting the input data for the AI tool [21], and the procedure for assessing and handling poor-quality or unavailable input data [21] through a review process. As an example, the authors in [23] developed a user interface to help both pathologists and AI scientists to create annotations and collect them without going through scripting. Examples of annotations are positive lymphocytes, tumor area, invasive tumor margin…etc. The type of annotations will be guided mainly by the objectives of the project [24]. Annotations can also be marked at different levels of detail; case-level annotations consist of a single label that can be assigned to a case and its representative slides or to each slide separately. The label can be binary (such as benign vs malignant) or multi-class (e.g., grade 1, 2, 3). These labels represent the overall diagnosis or prognosis. Region level annotations include different regions in the WSI which can be of any diagnostic or prognostic value for a project at hand. Finally, cell-level annotations are the more detailed annotations in which different cell types are either annotated by a point/dotting (for cell detection and classification), or by marking the boundary of nuclei in a free-hand manner [24].

#### 2.3.4. Preprocessing

**Figure 2:**
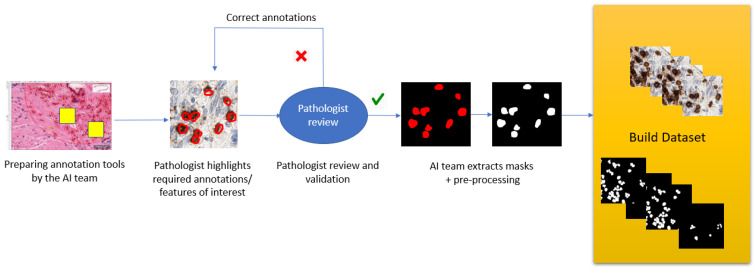
Example of an annotation process and building the final dataset.

Preprocessing brings annotated regions within the slides in a format that is recognizable by a machine learning/ deep learning algorithm. It includes extracting the dataset in the form of patches, adjusting the patch size to the model input size, and applying the appropriate image conversion, often binary or logical format (as shown in Figure 2). With slides coming from different laboratories, there should be consideration given to variability between them, addressing this, using techniques such as image normalization [25] which is a typical process used in the preparation of a dataset for AI. This method puts multiple images into a common statistical distribution in terms of size and pixel values; i.e., intensity normalization would convert an input image into a range of pixel values that are more familiar or normal to the senses, hence the term normalization. The pathologist’s knowledge and expertise, benefit the process of setting this normal range by choosing for example slides from the different laboratories. As an example, a normalization based on prior knowledge of the healthy prostate tissue intensities may be the most effective way of acquiring the desired properties of normalized images [26].

Other common issues include artifacts and batch effects, unintentionally introduced during both routine slide preparation such as precipitates of staining dyes and tissue folding, and digitization where there may be a loos in focus, blurriness, or variations in contrast and hue. To ease the process of laborious manual review of glass and digital slides for pathologists, and given the fact that this task can be qualitative and subject to intra- and inter-reader variability, pathologists can make use of helpful tools such as HistoQC [27]. Such a tool rapidly performs quality control, and not only identifies and delineates artifacts but also discovers cohort-level outliers where slides may be stained darker or lighter.

#### 2.3.5. Data Augmentation

To boost the lack of data during the data preparation step, and to increase the generalization capabilities of their algorithms, AI scientists apply data augmentation, which is the process of generating new image patches from originals, using random rotation, flip, brightness, random shift, and more recently proposed, adversarial neural networks GANs [28].

### 2.4. Model Design and Training

**Figure 3:**
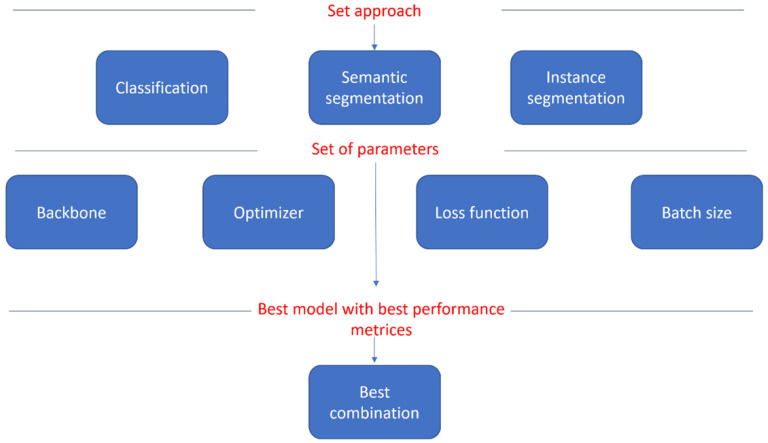
Best model selection process.

Understanding the type of outcome the pathologist expects from the AI tool, helps the AI scientists to select the right approach to characterize the slide. Examples of deep learning models that categorize selection based on the problem include classification, semantic segmentation, instance segmentation, spatial relationships, regression, and many others, dependent upon the intended use of the AI algorithm. A method that our group proposed has shown to be efficient [23] proposes that the AI scientist goes through sets of different model parameters testing; this encompasses applying different backbones, optimizers, loss functions, and batch sizes, and finally finding the best combination of those that result in the best performance metrics (Figure 3). We presented a working model which describes this process in details [23]. Performance metrics should also be selected carefully, such as accuracy, sensitivity, specificity, Jaccard index, precision, and recall, and there may be different levels at which they are measured, such as pixel-level, object-level, or case/slide-level. These are indicators for the AI scientist regarding the performance of the model. Clinical metrics will have to be evaluated with the pathologist. If for example, the model performs positive cell segmentation and density estimation, the pathologist needs the object level precision and recall, where an object represents a cell, rather than the pixel level values for the same metrics. This performance still needs to be validated by a pathologist, through the application of the algorithm on a test dataset never seen by the model during training, with the results presented to the pathologist for review. The advice of the pathologists is key in helping the AI scientist to estimate the degree of acceptable error reported. This will also inform where the model’s parameters and performance output can be maximized through data set selection.

### 2.5. Clinical Utility and Hypothesis Validation

The outcome from the design and development of the AI algorithm remains a way to validate the hypothesis or to explore any clinical significance of the results in relation to the patient. Depending on the application, pathologists may require further analysis of the output of the AI models (statistical, survival, and so on) to get a better understanding of the patient and the way it can be used to develop a tool for use in the clinical domain.

## 3. Deployment

**Figure 4:**
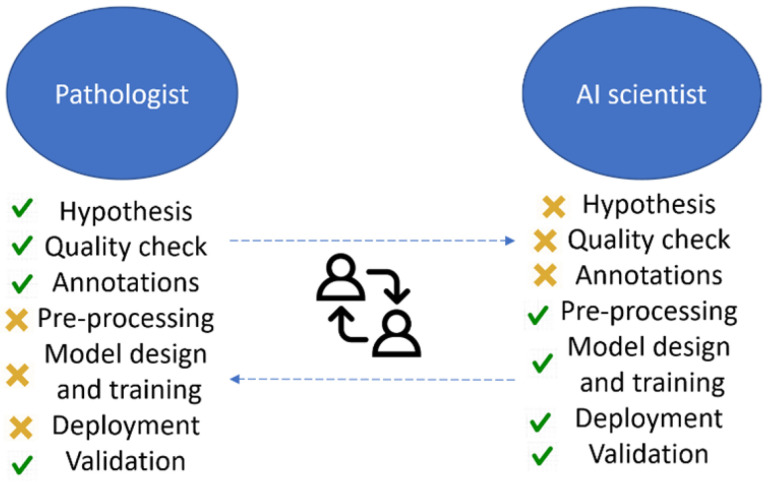
Tasks performed by pathologists and AI scientists.

Before deployment, it is important to discuss with the pathologist how interaction with the AI tool will occur, what they want to control in the slide image, such as region selection, and what needs to be displayed as a result. The AI tool is not just the algorithm, but the algorithm embedded within a workflow with associated visualizations and user experience. As an example, the pathologist selects a region of interest, and the AI tool displays the density of positive biomarker cells within that region. The pathologist may prefer to display the density along with a group classification so that they still have control and a better understanding of the data. A simple demonstrative interface can be very useful to set up all the necessary parts of the tool before proceeding to the software development. The logistics of incorporating AI diagnosis flow considerations are encountered when the pathologist undergoes a validation in the use of the AI tool, the workflow of which will be specific to the clinical question. This will also include software engineering factors such as the user experience and user interface (UX/UI) as well as the workflow for the AI product within the laboratory. These will require input from stakeholders, especially the pathologists. Robust performance validation of any AI tool will require an analytical validation in the delivery of the tool to include performance metrics such as reproducibility and precision, a clinical validation in the use of the AI tool to include accuracy and specificity, and the validation of the pathologist in use of the tool in addressing the patient-oriented question the AI tool is addressing, using the current means of assessment as a ground truth. Such validation is a requirement for approval by regulatory bodies and should be designed and specified in collaboration with those regulatory bodies and clinicians before they can be considered for use in clinical practice. Upon validation, the tool will be ready to use and can go further with regulatory body testing. Figure 4 summarizes the main tasks performed by the pathologist’s team and the AI scientist’s team. It is important to note that collaboration is present in every step. In a time of budgetary constraints, value for money or what added value an AI tool delivers to the clinical question needs to be addressed, often with procurement departmental oversight. Finally, the return on investment will depend on whether the healthcare system is a universal healthcare system. Such considerations have been dealt with in delivering novel molecular services in universal healthcare, and the same principles apply to the delivery of AI tools [29].

## 4. Conclusions

In this paper, we covered a walk-through scenario describing the collaborative work between pathologists and AI developers towards the design and development of an AI tool in pathology, particularly where there is a high level of supervised learning, and depending on the pathologist for ground truth values. We have shown that a pathologist’s knowledge and expertise are required and should be present at every step, from hypothesis formulation, through data preparation, model development, and validation to AI tool deployment. Throughout the process, the data scientist must curate and maintain a database of images, and where they were used in the training, or testing process. The process of concept exchange with the pathologist entirely determines the approach and tools that AI scientists should use to build a solid and reliable tool that provides improved patient outcomes whether it is scoring, diagnostic, or any other clinical output.

## Data Availability

Not applicable.
